# Significance of Coronary Revascularization for Coronary-Artery Obstructive Lesions Due to Kawasaki Disease

**DOI:** 10.3390/children6020016

**Published:** 2019-01-29

**Authors:** Soichiro Kitamura, Etsuko Tsuda

**Affiliations:** 1 Department of Cardiovascular Surgery, National Cerebral and Cardiovascular Center, Suita, Osaka 565-8565, Japan; 2 Department of Pediatric Cardiology, National Cerebral and Cardiovascular Center, Suita, Osaka 565-8565, Japan; etsuda@ncvc.go.jp

**Keywords:** Kawasaki disease, coronary-artery bypass grafting (CABG), percutaneous coronary intervention (PCI), coronary-artery aneurysm, internal thoracic artery, myocardial infarction

## Abstract

As an acquired ischemic heart disease in childhood, coronary-artery disease caused by Kawasaki disease (KD) has been known worldwide since the mid-1970s. KD patients who develop coronary-artery obstructive disease often need revascularization some time in their life. Coronary-artery revascularization for KD coronary lesions can be done with the surgical coronary-artery bypass grafting (CABG) and percutaneous coronary intervention (PCI) procedures. However, the characteristics of coronary-arterial lesions caused by KD significantly differ from atherosclerotic coronary disease in adults. Therefore, it is much more difficult to determine the optimal time and selection of a coronary-artery revascularization procedure for KD sequelae. CABG using the internal thoracic artery has been accepted as a very useful and beneficial procedure since the mid-1980s, even in small children. Although the use of PCI in the late period can be effective in some adolescent and adult patients, the small vessel size and severe coronary-artery calcification are often limiting factors for its use in children. Therefore, CABG is a better approach for severe leftanterior descending artery and multiple-vessel disease in children and adolescents with KD coronary sequelae. Good coronary revascularization can improve the long-term outcomes of patients with severe KD complications.

## 1. Introduction

Kawasaki disease (KD) has become one of the most widespread acquired heart diseases worldwide in children over the last fifty years since it was first reported in 1967. The first national KD survey in 1970 in Japan indicated coronary-artery involvement in about 10% of affected children and 1.4% of cardiac deaths [1]. Recently, the incidence of coronary-artery aneurysms decreased due to the treatment of acute KD vasculitis with high-dose intravenous immunoglobulins. Nevertheless, at present, about 0.2%–0.3% of Japanese KD patients develop giant coronary-artery aneurysms. These post-KD patients develop ischemic heart disease due to coronary-artery stenosis over a long period of time [2,3]. The number of patients needing coronary revascularization is very small in this population, and reports of percutaneous coronary intervention (PCI) and coronary-artery bypass grafting (CABG) in this field are not abundant, particularly in Western countries [4]. KD patients are generally smaller and younger than adults, with coronary-artery disease caused by atherosclerosis, and coronary-artery lesions caused by KD have many different characteristics, such as progression and/or regression with morphological changes over time, and good ability for collateral formation in children. Therefore, this condition presents various challenges that coronary revascularization in adults does not. Here, we describe the current role of coronary revascularization in patients with coronary-artery lesions due to KD. 

## 2. Coronary-Artery Lesions Caused by KD

Coronary-artery aneurysms change in morphology over many years. Acute myocardial infarction often occurs in patients with giant aneurysms (≥8 mm in diameter) within the first year of onset of acute KD, which leads to left ventricular dysfunction or sudden death [5,6]. Severe localized stenosis due to coronary wall thickening after KD can cause myocardial ischemia [7]. Coronary-artery calcification, one of the characteristic lesions caused by KD, is also found in the late stages of the most severe cases of localized stenosis [8]. The time of coronary-artery occlusion varies for each coronary artery in each patient. Furthermore, the larger the diameter of the maximum coronary-artery aneurysm, the more extensive and bilateral the coronary-artery aneurysms [9]. Therefore, the occurrence of a giant aneurysm is often indicative of a multivessel disease. Myocardial infarction in patients with bilateral giant aneurysms has a strong effect on survival outcomes in the early and late stages after the onset of KD. However, the occurrence of a giant aneurysm in the right coronary artery can sometimes asymptomatically cause occlusion, and the incidence of clinically significant lesions in the right coronary artery is much lower than in the left anterior descending artery because of its good ability to form intracoronary and intercoronary collateral vessels. 

**Figure 1:**
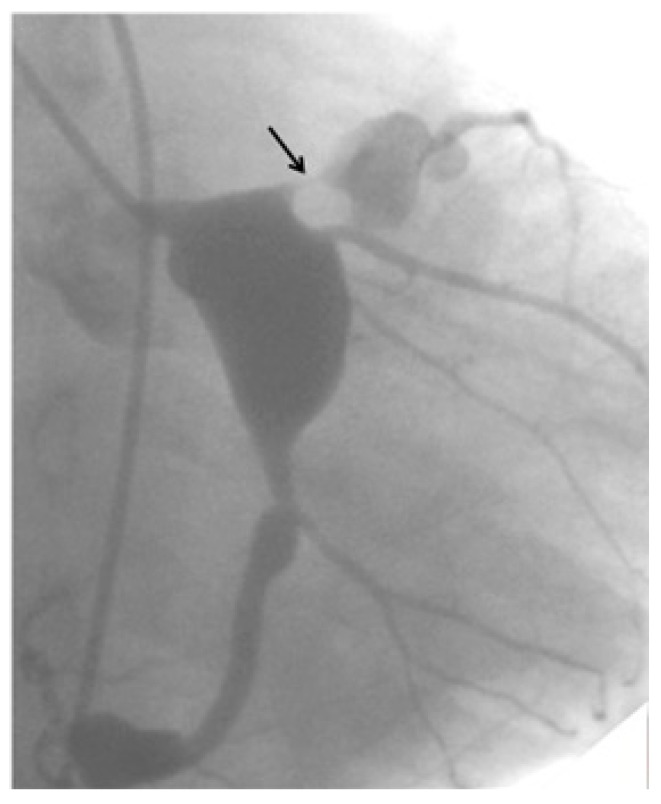
Selective coronary angiogram of the left coronary artery before operation. A seven-year-old Serbian girl who had had Kawasaki disease (KD) at infancy developed localized tight stenosis just at the proximal portion of the left anterior descending artery.

**Figure 2:**
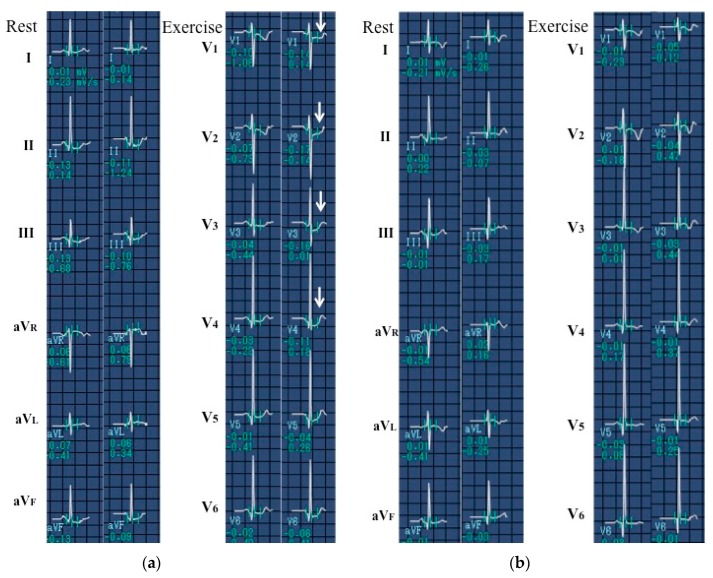
Findings of the treadmill testing before and after surgery. (**a**) Before surgery. During exercise, a seven-year-old Serbian girl had ST-T depression in leads V_1–4_ due to severe localized stenosis of the left anterior descending artery, as shown in Figure 1. (**b**) After surgery. ST-T depression in leads V_1–4_ disappeared after internal thoracic artery (ITA) grafting to the left anterior descending artery, as shown in Figure 3.

When severe localized stenosis is detected in coronary angiography, either a treadmill test or radioisotope stress myocardial perfusion imaging should be performed (Figure 1, left; Figure 2) [10]. Measurements of coronary-flow reserve by transthoracic echocardiography may be useful to determine whether there is any reduced coronary-artery flow due to coronary-artery stenosis [11]. However, even when significant localized stenosis is detected in coronary angiography, patients are often asymptomatic, and the onset of acute myocardial infarction can be the first event. In addition, judgment regarding the grade of stenosis is not always easy because of the presence of nearby aneurysms and dilations of the coronary artery. Recently, the use of fractional flow reserve (FFR) has been recommended in atherosclerotic coronary disease [12]. However, the value of an FFR study in KD coronary disease in children is not certain; a more precise method to detect coronary narrowing is needed in patients with combined coronary aneurysmal and obstructive lesions that are the specific features of KD coronary disease [12,13]. Recently, new diagnostic technologies have been applied to (KD) coronary-artery disease, namely, transluminal attenuation gradient (TAG) analysis of computed tomography angiograms [14], and optical coherence tomography (OTG) [15]. Both methods can be good armamentaria in the evaluation of the coronary-artery wall structure and resultant hemodynamic abnormality. However, at the present time, these methods are not routinely used for grade judgment of coronary obstructive lesions due to KD.

## 3. Choice of Methods for Coronary Revascularization

The first role of coronary revascularization is to improve myocardial ischemia, which can consequently preserve left ventricular function. The second role is to improve the long-term prognosis of KD patients with coronary-artery lesions. Indications for the coronary revascularization procedure have been reported in treatment guidelines from America [10], Europe [16], and Japan [17]. It is important and sometimes difficult to select the best approach—either CABG or PCI. Grafting provides a new route to restore myocardial blood flow. On the other hand, the target of revascularization by PCI is the diseased native coronary artery, which consequently often results in the recurrence of stenosis, requiring reintervention [4,18]. Therefore, both methods of revascularization fundamentally differ. Body size and the presence of a multivessel disease are also important factors in the selection of the appropriate coronary-revascularization methodology [19].

CABG is very useful in patients with a multivessel disease and severe left anterior descending artery disease [19,20]. In this population, CABG is a better approach for coronary revascularization than PCI, particularly when patients are small children [20]. Although PCI may improve myocardial ischemia for localized stenosis with giant aneurysms, the existence of giant aneurysms does not prevent thrombotic occlusion, resulting in myocardial infarction. On the other hand, when a good flow through the internal thoracic artery (ITA) is confirmed after surgery, the graft prevents distal thrombotic or embolic infarction [20]. Consequently, the risk to quality of life is lower with CABG.

The surgical indication for patients with severe delayed coronary flow through giant aneurysms is difficult. When ITA bypass surgery is conducted for insignificant stenotic coronary arteries, the graft undergoes diffuse thinning (string phenomenon). This occurs without symptoms, and although in some cases the ITA graft can recanalize well and become functional following the progression of the stenostic lesion [20,21,22], most string ITA grafts undergo thrombosis. Therefore, the use of CABG for giant aneurysms without significant localized stenosis should be deferred and performed under medical surveillance. As the presence of correct indications and the appropriate timing of CABG surgery after KD greatly contribute to graft patency, patients should be closely followed up, and CABG may be deferred until clear indications develop [16,17]. 

The selection of whether CABG or PCI is suitable for a particular patient must be individualized based on perceived benefits and potential disadvantages due to complications. The usefulness of CABG has also been reported in some patients with coronary-artery abnormalities and coronary-transfer complications in congenital heart disease [23], and CABG utilizing the ITA graft can be performed at any age from the infantile period [23,24]. Both PCI and CABG are most probably required in patients who have a multivessel disease over a long lifespan of more than 50 years, from childhood to adulthood. 

## 4. CABG

### 4.1. CABG for Coronary-Artery Stenotic Lesions after KD

The use of CABG for KD coronary sequelae was first reported in 1975 by Kitamura and coworkers who utilized saphenous vein grafts (SVG) in children [25]. Furthermore, CABG using a single or bilateral ITA was also shown to be successful in 1983 by Kitamura, who reported the safety and benefits of the procedure for children [26,27]. The gastroepiploic artery (GEA) and radial artery were then used in 1987 and 1996, respectively [28,29]. The importance of arterial grafts in CABG for children stems from the apparent difference in patency rate for arterial and venous grafts on a long-term basis [20,30,31,32]. Most of the time, arterial grafts, particularly pedicled ones, are utilized in growing children, and we believe that venous grafts should be avoided for children [20]. The number of CABG procedures for KD coronary-artery lesions increased during the 1990s [29], and at the time of writing this article, the annual number of CABG is about 30 patients per year in Japan [33]. The prevalence of CABG based on the annual number of new KD patients in Japan is about 0.3%, and this is about one-third of the prevalence of patients who develop giant aneurysms [34].

CABG operations in children are performed under cardiopulmonary bypass and aortic cross-clamping with cardioplegic myocardial protection. The ITA is thin-walled and has a diameter of about 1 mm in small children. Therefore, the procedure requires careful anastomosis under high-magnification surgical glasses or a microscope with size 8-0 to 9-0 small needles and sutures [20,35,36]. In young adults, off-pump CABG is possible [37] through minimum intercostal incision with the aid of robotic instruments for ITA dissection.

**Figure 3:**
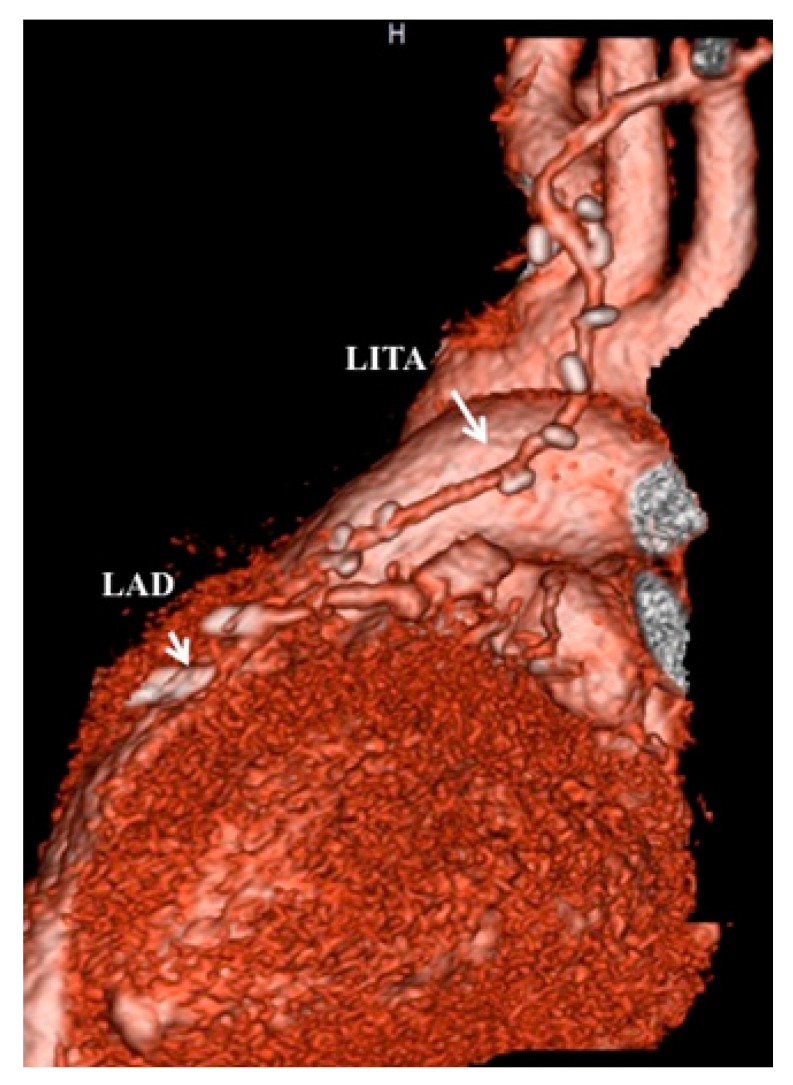
A computed tomography angiogram 10 days after coronary-artery bypass grafting revealed a well-patented ITA graft to the left anterior descending artery (same patient as shown in Figure 1 and Figure 2).

**Figure 4:**
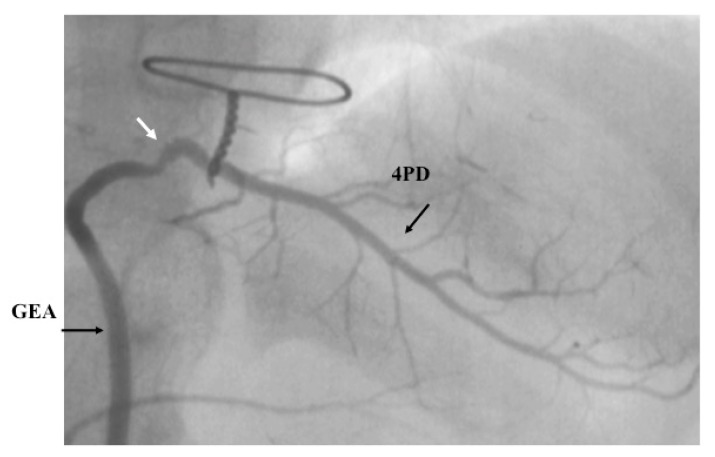
Gastroepiploic-artery (GEA) graft 11 months after surgery. A six-year-old boy had grafting of the posterior descending branch of the right coronary artery with GEA.

Although CABG with SVGs was performed in children in the 1970s–1980s, the patency of the SVG was not satisfactory [20,30,31,32]. Acute myocardial infarction occasionally occurred due to thrombi from the degenerated SVG in the late postoperative period [31]. In general, the SVG did not grow in proportion to somatic growth. On the other hand, pedicled arterial grafts allow for longitudinal and circumferential growth, have good long-term patency, and are thus preferred in the young and growing population [20,32,36,38,39] as shown in Fig.3. However, they have limitations in terms of number and length. Specifically, it is difficult to anastomose an ITA graft alone to the posterior descending branch of the coronary artery because of its limited length. To solve this problem, the GEA can be used to bypass the distal right coronary artery in children (Figure 4). When the number and length of the graft are limited, sequential grafting or a combination graft of the ITA with the radial artery can be used for reoperations in adulthood [37].

### 4.2. Long-Term Results of CABG in Children with KD

#### 4.2.1. Graft Patency and Long-Term Surgery Outcome

**Figure 5:**
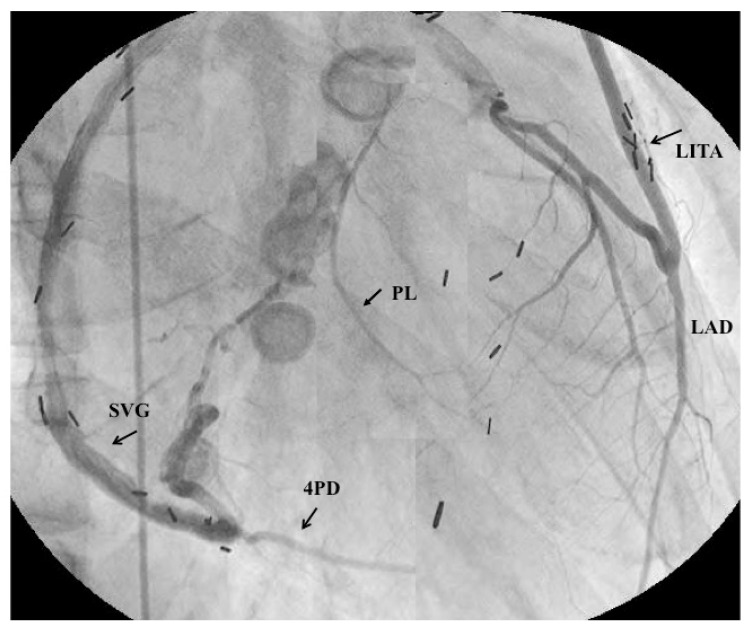
Selective angiograms 34 years after coronary-artery bypass grafting (CABG). This male patient had surgery at the age of six years old. The saphenous vein graft and internal thoracic-artery graft demonstrated patency. Both the left main coronary artery and the right coronary orifice were asymptomatically occluded during the late period after the surgery (this was the first case to use an ITA in a child) [26].

A previous study by us [32] found the 25-year patency to be 87% (*n* = 154, 95% CI 78–93) for ITA grafts, and 44% (*n* = 30, 95% CI 26–61) for SVG. The patency difference between ITAs and SVGs was significant at the *p* < 0.001 level. Although the patency rate for SVGs was lower (25%), particularly when used in small children (*n* = 12, 95% CI 6–51), some patients demonstrated patency lasting over 30 years, as shown in Figure 5. This particular patient simultaneously had a prosthetic mitral-valve replacement and had thus been on warfarin therapy for a long time, which may have contributed to long-term SVG patency.

However, ITA grafts in small children have previously been problematic due to their link to anastomotic stenosis, mostly as a result of technical reasons that develop soon after operation. Anastomotic stenosis during the early postoperative period can be treated by one-time balloon dilation [20,24], and recurrence is rare [31]. A recent series, however, showed that there was no significant difference (*p* = 0.163) in the patency rate of ITA grafts between children aged less than 10 years old (*n* = 66, 86%, 95% CI 74–93) and those aged more than 11 years (*n* = 88, 93%, 95% CI 83–97) [32].

**Figure 6:**
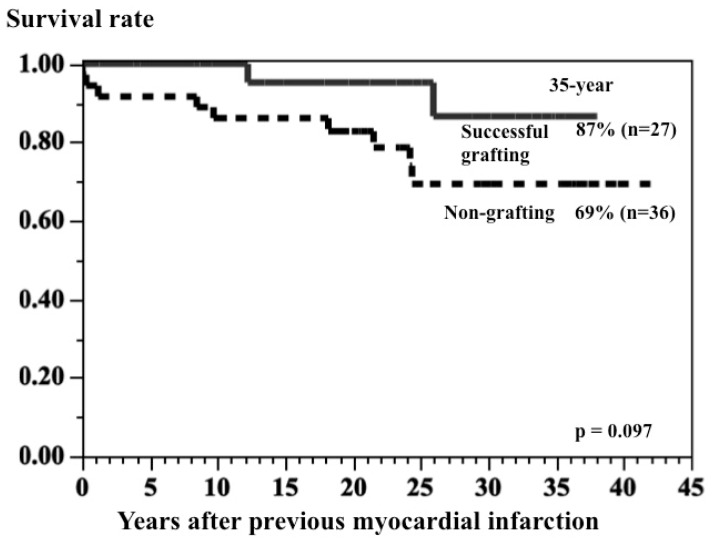
Comparison of survival rates of patients with previous myocardial infarction between the successful CABG group and nongrafting group.

Among the 114 patients who had undergone CABG when younger than 20 years of age in our institution [32], survival rate after 25 years was 95% (95% CI 89–98), and all 109 survivors were categorized as New York Heart Association Class I [32]. In patients with a previous myocardial infarction, survival rate after 30 years in patients with at least one successful bypass grafting was 87% (95% CI 57–97, *n* = 27), compared with 69% (95% CI 5–84, *n* = 36) in patients who did not undergo surgery; however, the difference did not reach statistical significance (*p* = 0.097) because of the small number of cases used in the comparison and because the groups were not matched (Figure 6) [40]. The outcome in patients after CABG was determined by graft patency and reduced left ventricular function (LVEF). When at least one graft was patent, particularly for the left anterior descending artery, and the LVEF was 50% or greater, the long-term outcome was good. In particular, good coronary revascularization to the left anterior descending artery produced a good long-term result [20,40].

#### 4.2.2. Cardiac Events after CABG

As the progression of coronary-artery stenosis in each branch is not always the same, either a second operation or PCI is often needed in adulthood. The cardiac event-free rate 25 years after the operation was 60% (95% CI 46–72) [32]. This means that long-term follow-ups are required in this group of patients. The survival and event-free rates are poor in patients with reduced left ventricular function and ventricular arrhythmias. Nonsustained ventricular tachycardia is a risk factor for sudden death in patients with a low LVEF [40]. Antiarrhythmic treatments are needed to prevent sudden death. Catheter ablation for ventricular tachycardia is successful in some patients, and the use of an implantable cardioverter defibrillator should also be considered. Furthermore, orthotopic heart transplantation becomes necessary for a few patients with advanced heart failure due to previous myocardial damage [41]. Either cardiac resynchronization therapy or a left ventricular assist device should also be considered in such patients while they are awaiting heart transplantation.

## 5. PCI

### 5.1. Percutaneous Transluminal Coronary Rotational Atherectomy (PTCRA)

**Figure 7:**
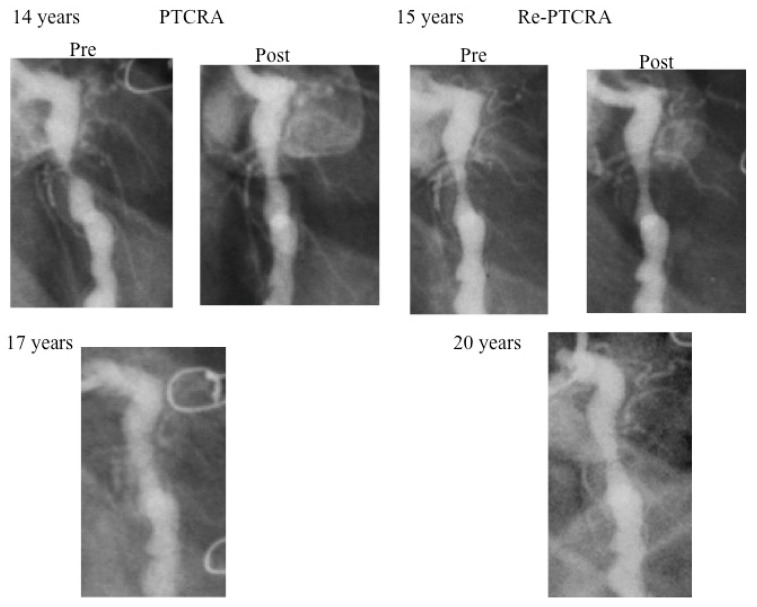
Changes in the degree of stenosis after percutaneous transluminal coronary rotational atherectomy (PTCRA). In a 15-year-old boy, good patency of the left circumflex was maintained after repercutaneous transluminal coronary rotational atherectomy (PTCRA).

The coronary arterial wall develops calcification over a long-term period. Therefore, rotational atherectomy is generally applied for severe coronary stenosis with calcification [42,43,44,45]. PTCRA is suitable for cases of short stenosis without giant aneurysms. A five-year-old boy is the youngest patient to have undergone successful PTCRA [43]. Although the result of this case initially appeared effective, asymptomatic occlusion occurred within a year after the procedure (Figure 7). The size of the burr used in small children is limited because of the small diameters of the access artery and the target vessel. The recurrence of fibrocalific stenosis is also high in this setting. Thus, PTCRA cannot always be recommended in small children. However, when a larger burr (>2.15 mm diameter) can be used, good patency of the vessel may be expected. Repeated rotational atherectomy is often needed for restenosis, especially within the first year (Figure 7) [43]. New aneurysms may appear when high-pressure balloon angioplasty is combined with PTCRA, and this combination of PCI should be very cautiously performed or avoided [44]. Knowing the long-term efficacy and complications of PTCRA is important in deciding the timing of this procedure. 

### 5.2. Percutaneous Transluminal Coronary Balloon Angioplasty (PTCBA)

The coronary-artery intima with fibrocellular proliferation is not firm in the early period after KD. Therefore, PTCBA is effective for severe localized stenosis occurring within 1–2 years after the onset of KD. PTCBA in children can be considered for cases with early-appearing localized stenosis without a giant aneurysm, but recurrence of stenosis must be carefully followed [45,46]. 

### 5.3. Limiting Factors of PCI for Coronary-Artery Stenotic Lesions after KD

There are two major issues regarding the use of PCI in children with coronary-artery lesions due to KD. First, guiding catheters that are specifically designed or modified for small children are needed. Second, complications of the PCI can sometimes be fatal. Both intra-aortic balloon-pumping and perctaneous cardiopulmonary support are not easy to quickly install during an emergency in small children. In contrast, the use of ITA–CABG for KD coronary-artery disease is quite safe, with no cases of mortality in more than 100 patients in our hospital [32].

Coronary-artery lesions caused by KD persist long into adulthood. Thus, there are undoubtedly many asymptomatic adult patients with coronary-artery lesions caused by KD who remain undiagnosed [2]. KD coronary-artery lesions are not always familiar to physicians, and this population is a very small group among ischemic heart-disease cases in adults. Coronary-stent implantations are an accepted procedure in adults; however, there have been reports about stent fractures, new aneurysm formation, and stent malapposition in patients with coronary-artery lesions caused by KD [2,47,48,49,50]. Generally, the results of coronary-stent implantation for arteries with severe coronary calcification complicated by diabetes mellitus are poorer than those of CABG, and the long-term outcome of stent implantation remains unknown for inflammatory coronary obstruction that often occurs with calcification. For young children, it may also be better to avoid stent implantation, as stent growth cannot be expected in children, and stent fractures due to progressive coronary-artery lesions often occur [32,51].

## 6. Conclusions

Over the last three decades, coronary revascularization procedures have been developed for patients with coronary-artery lesions caused by KD. The efficacy of PCI is often limited by the body size of patients and the presence of severe calcific coronary-artery lesions, and the recurrence of lesions following this procedure is high. Surgical revascularization utilizing a ITA graft is the best choice for small children and adolescents. The correct selection of treatment modalities for coronary revascularization is important, and must be considered on an individual basis by weighing the possible benefits and disadvantages of each procedure. Good long-lasting coronary revascularization improves the outcomes of patients with KD, a condition that completely differs in the genesis of disease mechanism from atherosclerotic coronary-artery disease in adulthood.
